# Energy efficiency, resilience to future climates and long-term sustainability: the role of the built environment

**DOI:** 10.1098/rsta.2009.0212

**Published:** 2010-03-13

**Authors:** M. J. Kelly

**Affiliations:** Department of Engineering, University of Cambridge, 9 J. J. Thomson Avenue, Cambridge CB3 0FA, UK

**Keywords:** energy, sustainability, carbon dioxide emissions, built environment

## Abstract

Just under half of all energy consumption in the UK today takes place indoors, and over a quarter within our homes. The challenges associated with energy security, climate change and sustainable consumption will be overcome or lost in our existing buildings. A background analysis, and the scale of the engineering challenge for the next three to four decades, is described in this paper.

## 1. Introduction

For the first time in history, we have now had 10 years of global wide-bandwidth communications, so that no one has the excuse of being ignorant of other people’s problems. It is interesting to observe the outcome that the popular and political discourse is dominated by global issues of a Malthusian character: overpopulation, resource depletion, environmental degradation, climate change, the downsides of both poverty and affluence, etc. (We could add the global financial chaos to this list.) The concerns are heightened by the increasing information from sensing systems of the perturbations caused to our global ecosystems by the actions of mankind.

There are three particular challenges that all relate to the existing built environment. In the UK, we consume nearly half our energy inside buildings. Because the UK is now a net importer of energy, and we have run down our ability to store natural gas since we have had the North Sea as our gasometer for 30 years, we are particularly susceptible to spot-price fluctuations, and our energy security is in the hands of foreign countries. To the extent that we can reduce energy consumption within existing buildings, we are helping to alleviate the problem of energy insecurity, now a matter of strategic national importance. At the same time, we are helping to ensure that a lesser future energy infrastructure would be needed. About 45 per cent of our carbon emissions come from buildings and 27 per cent from our homes. Furthermore, about 87 per cent of today’s buildings will still be here and forming about 70 per cent of the building stock in 2050. Reducing these emissions is essential in any attempt to mitigate against future climate change. Furthermore, any work on adapting to climate change will invoke a major makeover of the existing buildings to cope with extremes of weather, such as localized flooding or near-horizontal monsoon-like rain. Finally, it is estimated that our present living standards, if shared by all those on Earth, would require three planet Earths to source in a sustainable manner, i.e. our lifestyle is absolutely unsustainable in an era of global equality. We are going to have to move closer to a lifestyle that is more sustainable. Given this 45 per cent role, the triple challenge of energy security, climate resilience and sustainable consumption will be seen off, or succumbed to, by what happens in the coming decades to the existing built environment.

In recent years, we have been building new homes at about 1 per cent of the stock per year. These are planned to be more energy efficient, and so it is the existing stock, rather than the new build, that should be our primary focus, as it will be in this paper. In late 2008, the UK Government enshrined in law a target of an 80 per cent reduction in carbon emissions by 2050. Without a radical makeover of today’s buildings and a renewal of the energy infrastructure, together a civil engineering project on a scale never contemplated before in peacetime, this triple challenge will not be overcome.

## 2. The UK building stock

There are 22 million homes in the UK and about 5 million non-domestic buildings. About 7 million homes were built before 1900. Building peaked at 410 thousand in 1968, but was down to 141 thousand in 2000, very similar to the value in 1900. The life of our homes is very long—only about one house is demolished for every 10 new ones built. Semidetached housing at 4.9 million is 31 per cent of the total stock, followed by low-rise flats and detached houses. The UK has been an urban society for some time, and the population in urban areas increased from 77 to 89 per cent of the total during the twentieth century. In France, this ratio grew from 59 to 74 per cent in the period 1954–1990 alone. The UK is an outlier in Europe, with 68 per cent of homes in owner occupation (up from 10% in 1900), while private renting dropped from 89 to 10 per cent in the twentieth century. In 1999, 17 per cent of all homes were rented from local authorities and 5 per cent from housing associations, although the balance between the two had been shifting towards associations as a result of legislation. These statistics form the backdrop of ownership against which the policies for seeing off the triple challenge must be couched.

## 3. The periods 1990–2005 and 2005–2020

The carbon emissions from domestic buildings dropped by 4 per cent from 154 MtCO_2_e (=million tonnes of carbon dioxide equivalent) in 1990 to 147 MtCO_2_e in 2005. During that 15 year interval, there was a 10 per cent increase in house numbers, a 4 per cent increase in population, and a sharp rise from a very low base in the electricity consumed by electronic appliances for IT and entertainment (e.g. computers and plasma screens). The reduction in CO_2_ emissions might have been 10 per cent or more without these countervailing factors. This reduction came, in the main, from the recorded steady progress in measures to improve the thermal envelope in houses. We take the following basket of interventions: installing 3 inches or more of loft insulation, double glazing more than 60 per cent of the windows by area, draught-proofing over 60 per cent of rooms by volume, and installing cavity wall insulation where appropriate. In 1990, about 35 per cent of all houses already had this standard of insulation and were already capturing the energy-saving benefits, and this figure rose to about 65 per cent by 2005. At the current rate of installation, these measures will be exhausted by 2015, and they have a strictly limited capacity to drive further deep reductions in CO_2_ emissions. When we note that the 2008 Climate Change Bill sets a 24 per cent reduction target by 2022, we can see that the building sector is going to have to work on its existing stock to achieve *six* times the net reduction in carbon emissions in the current 15-year period, and we are already 30 per cent through this second period. We have indicated a limited capacity of the thermal envelope to contribute, unless there is a major R&D project to bring forward new thermal insulation materials and products, with new and more effective means of installation. This factor of six sets the scale of the challenge that faces us for housing, let alone any other part of the national infrastructure—non-domestic buildings, energy supplies, transport, etc.

## 4. Measures to 2050

There are four ways in principle by which the operation of buildings can contribute their full share of an 80 per cent reduction, and all are needed:

- new measures to improve the thermal envelope of buildings—materials, installation processes, controls, etc.;
- decarbonizing the National Grid and other sources of energy;
- improving the energy efficiency of appliances; and
- changes in personal attitudes and behaviour concerning profligate energy consumption.

Of these, only the second is widely accepted in the public debate, and measures are being taken in relation to renewable sources of energy, a nuclear rebuilding programme and the renewal of a more efficient National Grid. Hard data on the aggregate energy of computing are hard to come by, but anecdotal evidence of 5 per cent of US electricity being used in IT, especially for cooling, has been quoted.

Behaviour change is well documented as a cause of high energy consumption in buildings. Our concept of comfort has evolved over the last 40 years in an era of cheap energy. Those with childhoods before 1960 can remember houses where one room was heated at any time. Now whole houses are routinely heated, and the ambient temperature has risen from less than 19^°^C to 22^°^C. Public buildings are controlled to a temperature of typically 22±2^°^C all year round, and even in winter, the morning blast of energy from 06.00 to 10.00 is followed after 12.00 with chilling of the air for the rest of the day to remain within the specified bounds. At Eland House in London, the headquarters of the Department for Communities and Local Government, a small move to maintain the temperature at 21±2^°^C in winter and 23±2^°^C in summer is anticipated to reduce the heating bill by 9 per cent and the cooling bill by 5 per cent. Mr Koisume, Prime Minister of Japan in 2005, ordered that no public buildings should be cooled below 28^°^C or heated above 22^°^C. This has led to a revised dress code (‘cool biz’, where men do not wear jackets or ties in summer). What evidence I have seen points at most to a 2 per cent drop-off in the efficiency of call centre operators working under this wider temperature regime in Tokyo. Redefining the acceptable levels of comfort at work and at home will be one of the major areas for meeting the interim carbon reduction targets for 2020.

## 5. Further context: the building sector

The noble efforts of a few to ‘green’ their homes at great personal cost in terms of money and time are all very well, but these are not role models for the whole of society. If we think that we might have at most two chances to make a major intervention to any given home between now and 2050, we must scale up over the next few years to a steady state of 1 million houses each year being subject to a whole-house intervention. One will see energy, water, waste and air systems being upgraded to be more efficient. This is about double what is estimated to occur at present in terms of renovation, a much more limited activity. Do we have the capacity to expand the sector to what is needed? Anything less will not get us to where we want to be.

The real problem is that there is no retrofit market. The renovation market, such as it exists, is totally ‘balkanized’, with small firms or single traders offering limited services. Reality TV shows depict poor workmanship when some interventions are undertaken. There are many suppliers of different products, with no large market leaders. The many small players are keen to play a role, but all are looking for clear leadership—none are willing to risk their own businesses by going out alone and ahead on the ‘green’ agenda while others continue to cut corners on products and services.

There is a further structural problem that needs fixing. In recent years, much public and some private money has been committed to R&D towards solving the problems of energy inefficiency, climate change and sustainability. Funding agencies can be assured of a route to market of successful R&D in nuclear rebuild, renewable energy, and carbon capture and storage. Someone bidding to research on new external cladding materials cannot get the support from big players that do not exist, and that person is at a disadvantage. Indeed, there are some novel technologies sitting on the shelf for want of a clear order for 10^6^ pieces that would justify the tooling up for manufacture.

The new building sector is better off, with demanding targets of ‘zero-carbon’ new buildings by 2016 and 2018. The new materials and products are likely to be closely coupled to new methods of construction that will not be applicable to retrofitting existing buildings, which are constrained by older methods of construction and were designed in an era of cheaper energy.

## 6. Advice

Given the responsibility for building regulations and codes, and for planning, in my former role there have been five areas in which I have been advising my civil servant colleagues to move to get the existing building stock to play its full role in seeing off the triple challenge. The common theme is to appreciate the scale of the problem and start towards measures that solve the problems at the right scale.

(i) I think that further and higher education (FE and HE) should volunteer (or be tasked) and be funded to get their own estates to the 2050 targets for carbon emission reductions, with the concomitant increase in energy efficiency and a move towards sustainable consumption by 2035, to show the rest of the country the way. Campuses have buildings that are proxies for private dwellings, public buildings, offices and factories. Some of the brightest minds in engineering and psychology are on campus, and if they cannot succeed, who else can we expect to do so? We can inspire the students, who are the leaders of tomorrow, to participate. The skilled personnel needed for the transformation of existing buildings can be recruited and trained within the FE and HE sectors. There are over 100 universities and more than 400 FE colleges, and at least one within 30 miles of every citizen in England. The scale of the HE/FE sector is big enough to engage the building sector in bringing new products and services to market. Having universities in the lead of efforts to ‘save the planet’ is a natural extension of their recently acquired role as engines of local economic growth, and a possible source of large philanthropic funding to support the public sector and fees-based income. Knowledge exchange is a core skill of academics, and they can be articulate advocates of what works and ardent critics of what fails in the journey towards a new national built infrastructure. Many universities are doing experiments at present, but not at the scale needed to impact the whole country; but the universities would like to be in that position.

(ii) Between them, the health, education, defence, social housing and local government sectors spent of the order of £10 billion per annum on renovation. By working together and specifying aggressive improvements in the performance of future thermal materials, products and installation processes, and better and more efficient appliances, the public sector could use their financial muscle to create and drive the retrofit market, just as the California legislature drove the market for the reduction in vehicle emissions from the mid-1980s. In 10 years, the individual home owner would find only superior-performance products on the market and at competitive prices. There could even be council tax surcharges incurred by inferior-quality work.

(iii) Central government ambitions for the nation are actually delivered at a local level within local authorities. Few universities, companies, local authorities or other bodies who espouse their ‘green’ credentials have any vision that extends beyond 2015. I would like to see model trajectories developed at the local authority level that will tell us how (say) Cambridge, Manchester or London are going to work in each of the eight five-year periods from 2010 to 2050 to meet the 2050 targets and the interim ones. There is no need to rush at everything indiscriminately. Some model trajectories, engineering equivalents of the economic arguments of Stern, would add immensely to the quality of policy formation and action plans. We do not want to imitate the first-generation biofuel actions, which have made matters worse on several fronts, produced little or no carbon savings and caused unwarranted disruptions to other aspects of civilized life. One factor that might come to the fore is the relatively greater effectiveness of some energy-efficient measures if they are undertaken at a community level or at some stage involving several households. Another is how one might cope if there are radical changes to the supply of energy. If, in 20 years from now, natural gas is regarded as too precious to be used as a heating fuel, and is reserved as an industrial feedstock, how will the UK move smoothly on a national scale to other forms of energy? It is individual actions, integrated at the local authority level, that will most effectively capture the problems and opportunities over the next 40 years.

(iv) Over the last four decades, public attitudes and behaviour have changed with respect to wearing seatbelts in cars, not drinking and driving, and not smoking in public confined spaces. We have to reach a position where the profligate use of any forms of energy is considered deeply antisocial, and personal behaviour tends to exploit any technology interventions rather than circumvent them. A commonly accepted redefinition of comfort at home and at work is an essential first ingredient, taking a steer from, and extending, the Japanese initiative.

(v) The planning system in the UK is premised on the basis that applicants have to show that they are not offending local plans. This will have to change to a more permissive regime if the carbon reduction targets are to be met. With 15 per cent of buildings in the southwest of England being either listed or in conservation areas, wherein most current methods of saving energy (solar panels on roofs, double glazing, external cladding, etc.) are not allowed, we can admit defeat now. With the advent of near-horizontal rain as a part of future extremes of climate, such buildings will be defenceless and subject to accelerated ruin, exactly what is not intended by current legislation. We will not want the National Trust having to hand over buildings to English Heritage, which has a wider experience of handling ruins. We are going to have to change for the sake of our heritage. More widely, concerned citizens should be met on the presumption of permission for a wide range of approved energy-efficiency measures.

## 7. The scientific and technical challenges

The pursuit of clean energy sources is well rehearsed in public discourse. If someone could see the way to treble the capacity of the National Grid and supply it with clean electricity for (say) £1 trillion by 2035, the debates might be over. However, the engineering challenges suggest that this could not be delivered in time. Under such circumstances, every little bit helps a little, but every large bit helps more.

Materials of much greater thermal insulation (per unit thickness) must become available in quantity, with accompanying properties of mechanical and other stability, and ease of application to both the interior and exterior walls of our buildings, including floors.

Better heat exchangers can reduce waste when air and water leave buildings. Civilized people can live with much less than half our present daily consumption of water—at present, 50 per cent of the UK water consumption is for domestic purposes, and the natural environment would improve if we drew less water from aquifers and rivers.

An IT revolution applied to the simplification of building controls could make deep inroads into energy consumption in buildings if carried through with new conventions on comfort. If more of our tasks could be undertaken virtually rather than physically, a smaller environmental impact might follow.

A major drive to reduce the energy consumption of appliances should put a much greater emphasis on the appliances themselves to reduce their energy needs rather than exhort users to use them more sparingly. Normally off, rather than normally on standby, is one early move.

For the R&D community, a new emphasis on the urgency of exploitation will be needed. The linkages between ultimate end-users and initial researchers will need to be closer than in the past. When sums of the order of £1 billion are devoted to UK programmes such as the Energy Technologies Institute, or Living with Environmental Change, those committing such sums are entitled not only to expect more than the outputs of 1000 independent £1 million programmes, but also to be able to say, within a decade, what difference such investment has actually made to the nation in practical change. The intermediary organizations that perform the linkages will come to play an even more important role. The Haldane principle leaves it to the scientists to decide what programmes to fund out of the public funding of science. Where a challenge to the nation can be considered at hand, the relative ease in implementation, and the likely impact at the large scale of implementation, will become a much greater determinant of how scarce R&D resources are deployed.

## 8. Conclusion

I know of no previous era where a global problem, or in our case now a set of global problems, has come to the fore with a timeline of three to four decades for making serious inroads. Whereas in 1900 half of UK public expenditure was on defence, by 2000 half of the public expenditure was on health and welfare. The cost to retrofit just our homes will be of the order of £1 trillion (at approx. £50 thousand for each of 22 million homes over the next 40 years), with a comparable sum spent on renewing the infrastructure of energy, water, waste and other supplies and disposals. Even 1 year ago, such sums would have been considered daunting, but £1 trillion has entered the public consciousness with the attempts to prevent the collapse of the banking system. If we soon see a sixfold increase in the rate of improvement of energy consumption of buildings in the current 15 years to 2020, compared with the period 1990–2005 above, we may continue with the heightened sense of urgency. If not, the cries for a Manhattan-style project, or the move by non-democratic bodies to launch geo-engineering projects, will gather force.

